# Voltage-Gated Potassium Channels as Regulators of Cell Death

**DOI:** 10.3389/fcell.2020.611853

**Published:** 2020-12-14

**Authors:** Magdalena Bachmann, Weiwei Li, Michael J. Edwards, Syed A. Ahmad, Sameer Patel, Ildiko Szabo, Erich Gulbins

**Affiliations:** ^1^Department of Biology, University of Padova, Padua, Italy; ^2^Department of Surgery, Medical School, University of Cincinnati, Cincinnati, OH, United States; ^3^Consiglio Nazionale delle Ricerche Institute of Neuroscience, Padua, Italy; ^4^Department of Molecular Biology, University of Duisburg-Essen, Essen, Germany

**Keywords:** Kv-channels, cancer, cell proliferation, mitochondria, cell death

## Abstract

Ion channels allow the flux of specific ions across biological membranes, thereby determining ion homeostasis within the cells. Voltage-gated potassium-selective ion channels crucially contribute to the setting of the plasma membrane potential, to volume regulation and to the physiologically relevant modulation of intracellular potassium concentration. In turn, these factors affect cell cycle progression, proliferation and apoptosis. The present review summarizes our current knowledge about the involvement of various voltage-gated channels of the Kv family in the above processes and discusses the possibility of their pharmacological targeting in the context of cancer with special emphasis on Kv1.1, Kv1.3, Kv1.5, Kv2.1, Kv10.1, and Kv11.1.

## Introduction

Potassium channels allow the flux of K^+^ down the electrochemical gradient across the plasma membrane as well as across membranes of intracellular organelles. Among the different types of K^+^ channels encoded by more than 80 genes, the voltage-gated potassium channels represent the largest and most complex family with numerous members, classified into Kv1-Kv12 subfamilies (Alexander et al., 2017). Most subfamilies have distinct channels (e.g., Kv1.1-Kv1.8, Kv2.1-Kv2.2, Kv3.1-Kv3.4, etc.) based on their different biophysical properties and pharmacological profile (Gutman et al., 2005; González et al., 2012). Kv5.1, Kv6s, Kv8s, and Kv9s encode subunits that have a regulatory role and are unable to form homotetramers on their own. Functional channel-forming alpha subunits harboring six transmembrane segments have been associated with different functions, ranging from ensuring cell excitability to the regulation of cell cycle. These proteins, expressed in different tissues, belong to six subfamilies named Kv1 (Shaker), Kv2 (Shab), Kv3 (Shaw), Kv4 (Shal), Kv7 (of which Kv7.1 is named KvLQT), and ether a go-go (EAG; Kv10, Kv11, Kv12). In some cases, members of a given subfamily can heteromultimerize to give a functional channel composed of four alpha subunits. Frequent alternate splicing and association with regulatory beta subunits further contributes to the enormous diversity of potassium channel functions. The crucial role of Kv channels in cellular and organ physiology is illustrated by the vast number of pathologies linked to their mutation [e.g., the long QT syndrome for Kv11.1 and Kv7.1 (Brewer et al., 2020), non-syndromic sensorineural deafness type 2 for Kv7.4 (Xia et al., 2020), etc.].

In addition to the Kv subfamilies whose members mediate efflux of potassium from the cell at depolarizing potentials (outward rectifiers), the so-called inward rectifiers (Kir) contribute to cells physiology by allowing greater influx than efflux of potassium ions at a comparable driving force. While Kv voltage-dependent potassium channels display an intrinsic gating thanks to the positively charged S4 transmembrane segment, inward rectification is caused by voltage-dependent block of the channel pore by cytoplasmic ions, including Mg^2+^ and polyamines. In addition, some of the calcium-dependent potassium channel family members (e.g., Big-conductance Ca^2+^-dependent K^+^ channel BKCa) are also activated by changes in membrane potential in addition of changes in intracellular [Ca^2+^] (González et al., 2012). Some members of the two-pore K^+^ channel family (e.g., some members of the TASK family) also show a slight voltage-dependence of their probability of being open (Enyedi and Czirjak, 2010).

In this review we will focus our attention on the members of the classical voltage-gated Kv channel family, who were among the first channels to be linked to the regulation of cell cycle (DeCoursey et al., 1984; McKinnon and Ceredig, 1986; Arcangeli et al., 1993; Pardo et al., 1998) and cell death (Szabo et al., 1996; Gómez-Angelats et al., 2000), both processes instrumental to cancer progression (for recent reviews see e.g., Pardo and Stuhmer, 2014; Serrano-Novillo et al., 2019; Capatina et al., 2020). Indeed, dysregulated cell cycle leading to unlimited proliferation as well as apoptosis resistance are two hallmarks of cancer cells (Hanahan and Weinberg, 2000). While the notion that intracellular voltage-gated K^+^ channels are also linked to cancer is emerging (e.g., Bachmann et al., 2019; Peruzzo and Szabo, 2019; Teisseyre et al., 2019), the present review focuses on the Kv channels located in the plasma membrane. Table 1 summarizes the major findings discussed throughout this review about the role of different Kv channels in cell proliferation, apoptosis induction and tumor growth. Furthermore, voltage-gated potassium channels as possible targets for pharmacological cancer therapy are discussed.

**TABLE 1:** Targeting of Kv channels and its effects on cell death and proliferation.

Kv1.1	– Inhibition with 200 nM Dendrotoxin decreases preclonal expansion of thymocytes – Inhibition with 1-10 nM Dendrotoxin reduces proliferation of MCF-7 breast cancer cells – **Inhibition with 100 nM Dendrotoxin arrests gefitinib-resistant H-460 NSCLC cells at G1/S and reduces tumor growth** – **Enhanced expression protects neurons from cell death stimuli** – **Downregulation or inhibition with 50 μM Agitoxin-2 in retinal ganglion cells rescues neurodegeneration** – Inhibition with 50 μg/ml KAaH2 reduces proliferation of U-87 glioblastoma cells – Kv1.1 silencing decreased proliferation of HeLa cells	– Freedman et al., 1995 – Ouadid-Ahidouch et al., 2000 – Jeon et al., 2012 – Lee et al., 2003; Shen et al., 2009 – Koeberle et al., 2010 – Aissaoui et al., 2018 – Liu et al., 2019
Kv1.3	– Inhibition with 200 nM Charybdotoxin decreases preclonal expansion of thymocytes – **Knockdown or inhibition with 1 nM Margatoxin arrests A-549 lung adenocarcinoma cells at G1/S and reduces tumor growth** – Expression of Kv1.3 increases cell proliferation independently from ion conductance – **Inhibition with 0.1-5 μM PAP-1 reduces proliferation of smooth muscle cells** – Activation with 0.1-1 μM LJ101019C increases NK cell proliferation and progression through G1/S – **Downregulation or inhibition with 50 μM Margatoxin in retinal ganglion cells rescues neurodegeneration** – Downregulation of both Kv1.3 and Kv1.5 protects macrophages from staurosporine-induced cell death	– Freedman et al., 1995 – Jang et al., 2011a – Cidad et al., 2012; Jimenez-Perez et al., 2016 – Lasch et al., 2020 – Geng et al., 2020 – Koeberle et al., 2010 – Leanza et al., 2012b
Kv1.5	– Knockdown reduces proliferation, induces an arrest at G0/G1 and increases apoptosis of MG-63 osteosarcoma cells – Downregulation of both Kv1.5 and Kv1.3 protects macrophages from staurosporine-induced cell death – **Knockdown protects endothelial cells from palmitate-induced apoptosis** – Inhibition with 1 μM DPO-1 rescues the suppression of proliferation and induction of apoptosis mediated by apogenin in pulmonary artery smooth muscle cells – **Inhibition with 0.3-3 mg/kg DPO-1 rescues H_2_O_2_-induced endothelial cell apoptosis**	– Wu et al., 2015 – Leanza et al., 2012b – Du et al., 2017 – He Y. et al., 2020 – Chen et al., 2012
Kv2.1	– Inhibition with 0.2-1 μM Hanatoxin reduces proliferation of uterine cancer cells – Dominant-negative isoforms protect against DTDP-induced apoptosis of cortical neurons, while overexpression increases susceptibility – Inhibition with 3 μM 48F10 confers resistance to DTDP-induced cell death in cortical neurons – Inhibition with 1-20 μM 48F10 confers resistance to DTDP-induced cell death in enterocytes – Inhibition with 10 mM TEA or 30 μM Donezepil reduces oxygen-glucose deprivation-induced apoptosis of HEK293 cells – **Inhibition with 1 μM Ts15 reduces proliferation of T cells (Ts15 also inhibits Kv1.3 and Kv1.2)** – **Interfering with Kv2.1 membrane insertion by a syntaxin-1A-mimicking peptide ameliorates cell death in an ischemic stroke model** – **Inhibition with 20 mg/kg vindoline, 1–10 μM or 50 mg/kg SP6616 or 10 μM or 20 mg/kg ETA improves β-cell dysfunction and ameliorates hyperglycemia in diabetes models** – Silencing of Kv2.1 partner Kv9.3 arrested cell cycle in A549 lung carcinoma	– Suzuki and Takimoto, 2004 – Pal et al., 2003 – Zaks-Makhina et al., 2004 – Grishin et al., 2005 – Yuan et al., 2011 – Pucca et al., 2016 – Yeh et al., 2017 – Yao et al., 2013; Zhou et al., 2016, 2018 – Lee et al., 2015
Kv3.1	– **Inhibition with anti-Kv3.1 antibodies or downregulation reduces proliferation of oligodendrocyte progenitor cells**	– Tiwari-Woodruff et al., 2006
Kv3.4	– Increased expression under hypoxia increases proliferation of oral squamous cell carcinoma cells – Increased activity after radiotherapy arrests myeloid leukemia cells at G2/M due to hyperpolarization of the membrane	– Qian et al., 2019 – Palme et al., 2013
Kv7.4	– Inhibition with 10 μM Linopirdine increases cell death of spiral ganglion neurons	– Lv et al., 2010
Kv10.1	– **Blockage with a monoclonal antibody decreases proliferation of different cancer cell lines and inhibits breast and pancreatic tumor growth** – Inhibition with 10 μM Astemizole reduces cell proliferation and viability in a cervical cancer model – **Inhibition with 1–3 μM Astemizole synergistically enhances the anti-proliferative effects of calcitriol on breast cancer cells** – **Simultaneous knockdown of Kv10.1 and overexpression of TRAIL induces apoptosis of MG-63 osteosarcoma cells and reduces tumor growth** – **Inhibition with 5 μM Astemizole reduces HepG2 and HuH-7 hepatocellular carcinoma cell viability and proliferation and tumor growth** – **TRAIL-ligated antibodies sensitize chemoresistant MDA-MB-435S breast cancer cells to chemotherapeutics and reduce tumor growth** – Knockdown or inhibition with 5 μM Astemizole increases U-87MG glioblastoma sensitivity to Temozolomide – Knockdown or inhibition with 2.5-7.5 μM Astemizole increases the sensitivity of SH-SY5Y to rotenone-induced apoptosis – Inhibition with 7.5-9 μM Astemizole acts synergistically with Gefitinib to reduce proliferation and increase apoptosis of lung cancer cell lines – TRAIL-ligated nanobodies induce apoptosis in prostate and pancreatic cancer cells – **Inhibition with 1–100 μM or 15 mg/kg Procyanidin B1 inhibits proliferation and migration of liver cancer cells and hepatoma growth**	– Gomez-Varela et al., 2007 – Diaz et al., 2009 – García-Quiroz et al., 2012, 2014 – He et al., 2013 – de Guadalupe Chávez-López et al., 2015 – Hartung and Pardo, 2016 – Sales et al., 2016 – Horst et al., 2017 – de Guadalupe Chávez-López et al., 2017 – Hartung et al., 2020 – Na et al., 2020
Kv11.1	– Inhibition with 1 μM E-4031 reduces proliferation of uterine cancer cells and arrests cells at G0/G1 – Inhibition with 25–50 μM erythromycin increases the sensitivity of HT-29 colon carcinoma cells to chemotherapeutics and vincristine-induced G2/M arrest – Inhibition with 100 nM Cisapride arrests the cell cycle at G1/S and increases apoptosis of gastric cancer cells – **Inhibition with 5–20 μM E-4031, 20 μM WAY, 1 μM Sertindole, 100 μM erythromycin or 20 μM R-Roscovitine increases susceptibility of acute lymphoblastic leukemia cells to chemotherapeutics and 20 mg/kg E-4031 reduces leukemia burden *in vivo*** – Knockdown or inhibition with 20–40 μM Doxazosin arrests the cell cycle at G0/G1 and induces apoptosis in glioblastoma cells – **Inhibition with < 10 μM CD-160130 leads to growth arrest and apoptosis of leukemic cells and reduces tumor growth** – Antibodies fused to Doxorubicin-loaded nanoparticles increase PANC-01 pancreatic cancer cell death and growth arrest at G2/M – **Inhibition with 7 μM E-4031 concomitantly with KCa3.1 activation increases cisplatin-induced cell death and G2/M arrest in colorectal carcinoma cells and reduces tumor growth** – **Kv11.1 activity stimulates proliferation of esophaegal squamous cell carcinoma cells, knockdown reduces tumor growth** – Decreased expression after application of a miR-96 inhibitor arrests bladder cancer cells at G1 – **Activation with 50 μM or 6 mg/kg NS-1643 arrests proliferation of MDA-MB-231 breast cancer cells and reduces tumor growth and metastatic spread** – Knockdown or inhibition with 10–20 μM E-4031 reduces proliferation and increases apoptosis of MG-63 osteosarcoma cells, while activation with 5–10 μM PD 118057 increases proliferation – **Inhibition with 40–160 μM Clarithromycin induces a G2/M arrest and apoptosis of HCT-116 colorectal cancer cells and potentiates the effect of 5-FU *in vivo***	– Suzuki and Takimoto, 2004 – Chen et al., 2005 – Shao et al., 2005 – Pillozzi et al., 2011 – Bishopric et al., 2014 – Gasparoli et al., 2015 – Spadavecchia et al., 2016 – Pillozzi et al., 2018 – Wang et al., 2019 – Xu et al., 2018 – Fukushiro-Lopes et al., 2017; Breuer et al., 2019 – Zeng et al., 2016 – Petroni et al., 2020

*Studies listed in the text in which direct modulation of channel expression or function (or both) was used to study their involvement in cell cycle progression and apoptosis induction are summarized. Papers in which an in vivo approach was also used are highlighted in bold.*

## Voltage-Gated Potassium Channels and the Regulation of Cell Cycle and Proliferation

Cell proliferation plays a fundamental role during embryogenesis, tissue renewal and remodeling as well as wound healing. The process is tightly regulated, and aberrant cell cycle progression leads to pathological conditions such as cancer. Voltage-gated potassium channels are important contributors to the mechanisms that control cell division. Because the topic has been extensively reviewed in the last years, we will only briefly summarize by which means Kv channels control cell cycle progression and show some recent advances in the field. The interested reader is referred to some excellent reviews [for general coverage of the topic, for instance (Blackiston et al., 2009; Urrego et al., 2014; Liu and Wang, 2019); regarding cancer (Leanza et al., 2016; Rao et al., 2015; Serrano-Novillo et al., 2019)]. Among the various voltage-gated channels, the most abundant information regarding their role in cell cycle regulation/proliferation is available about Kv1.1, Kv1.3 (for recent reviews see Pérez-Garcìa et al., 2018; Teisseyre et al., 2019), Kv1.5, Kv10.1 (Urrego et al., 2017), and Kv11.1 (He S. et al., 2020).

**FIGURE 1:**
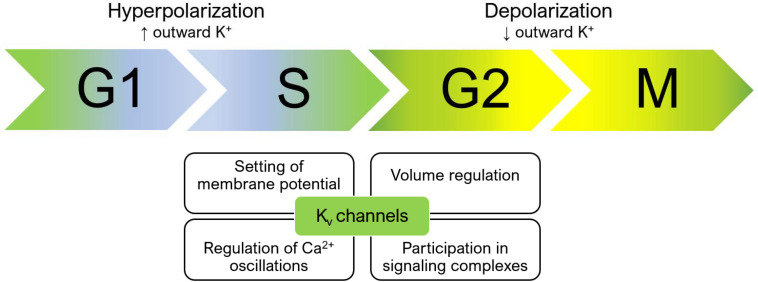
Membrane potential changes during cell cycle progression and the role of Kv channels. During the cell cycle, the membrane potential changes constantly. A hyperpolarization occurs during the transition from G1 to S, while the membrane depolarizes during the G2/M transition. Concomitantly, calcium oscillations in the cytoplasm and volume changes orchestrate the signaling events that occur at each of the different phases of the cell cycle. Kv channels regulate these processes by setting the membrane potential, thus influencing calcium influx and cell volume, and by participating in membrane signaling complexes. Accordingly, both their expression and function changes while cells proceed through the cycle. Please refer to the text for additional details.

Voltage-gated potassium channels regulate the progression through cell cycle checkpoints based on both their ion-conducting properties and their interaction with other proteins belonging to signaling complexes at the plasma membrane (Figure 1). The membrane potential is not constant during the cell cycle. At the transition between the G1 and S phase, the plasma membrane hyperpolarizes, while a depolarization is necessary for cells to proceed from G2 to M (Blackiston et al., 2009). Cells with a high proliferation rate tend to be less polarized at each step of the cell cycle. Consequently, cancer cells generally exhibit a depolarized phenotype (Pardo and Stuhmer, 2014; Serrano-Novillo et al., 2019). Potassium channels, as important contributors to setting the membrane potential, regulate cell cycle progression. Their role in mitogenesis was first proposed in 1984, when Cahalan and his team found that potassium channel blockers inhibited the proliferation of T lymphocytes (DeCoursey et al., 1984).

Kv channels influence cell cycle progression not only by setting the membrane potential, but also, as a consequence, by contributing to ensuring the driving force for calcium entry (for reviews see e.g., Cahalan and Chandy, 2009; Urrego et al., 2014) and the exit of chloride ions (and the resulting cell shrinkage). Accordingly, Kv channel expression changes during the progression through the different phases of the cell cycle, as first revealed more than 30 years ago in T lymphocyte development (McKinnon and Ceredig, 1986). Changes in the subcellular localization of different Kv channels also seem to play a relevant role (Serrano-Novillo et al., 2019). Independently from their ion-conducting properties, also the interaction of specific Kv channels with proteins mediating intracellular signaling cascades regulates cell cycle progression (Urrego et al., 2014; He S. et al., 2020).

### Kv1 Channels

As important regulators of cell proliferation, numerous reports have correlated the activity and expression of various potassium channels to the growth and progression of multiple kinds of cancers (recently reviewed in Serrano-Novillo et al., 2019). Kv1.1, along with Kv1.3, has been identified as a critical player to thymocyte pre-clonal expansion (Freedman et al., 1995). Later on, Kv1.1 was shown to be expressed in the breast cancer cell line MCF-7 and its blocker Dendrotoxin (DTX) (10 nM) reduced proliferation by 30% (Ouadid-Ahidouch et al., 2000). Likewise, DTX suppressed lung adenocarcinoma growth *in vivo* (Jang et al., 2011b) and at 100 nM concentration, it reduced proliferation of chemoresistant non-small cell lung cancer (NSCLC) cells both *in vitro* and *in vivo* (Jeon et al., 2012). Inhibition of Kv1.1 by KAaH2 toxin targeting specifically Kv1.1 was shown to inhibit proliferation of glioblastoma via the Epidermal Growth Factor Receptor (EGFR) signaling pathway (Aissaoui et al., 2018). In addition, Kv1.1’s high expression was observed to correlate with poor prognosis of cervical cancer patients and silencing of the channel blunted proliferation of HeLa cells (Liu et al., 2019).

**FIGURE 2:**
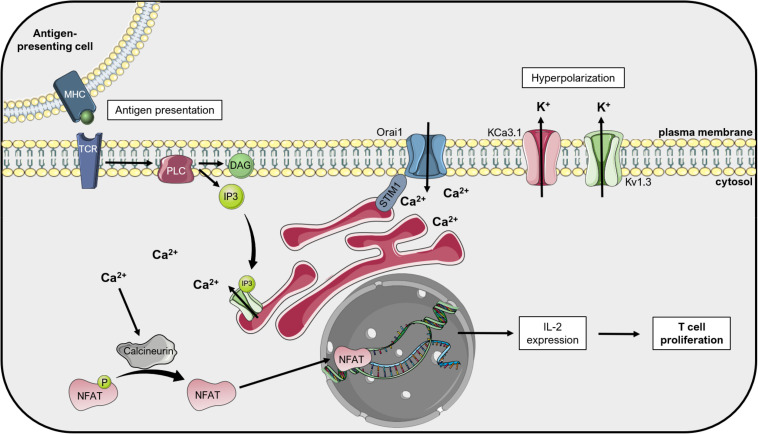
Kv1.3 regulates T lymphocyte proliferation: the «membrane potential model». Upon activation of T cell receptors (TCR) by antigen-presenting cells, phospholipase C (PLC) cleaves the phospholipid phosphatidylinositol 4,5-bisphosphate into diacylglycerol (DAG) and inositol 1,4,5-triphosphate (IP3). IP3 activates the IP3 receptor (IP3R) on the endoplasmic reticulum (ER), which releases Ca^2+^ into the cytosol. Ca^2+^ depletion from the ER lumen leads to conformational changes of the ER-resident protein STIM1, which couples the ER to the plasma membrane and activates Orai1, a calcium release-activated Ca^2+^ channel (CRAC). The following increased Ca^2+^ concentration in the cytosol activates the phosphatase Calcineurin, which dephosphorylates the Nuclear Factor of Activated T cells (NFAT). This transcription factor translocates to the nucleus and activates the transcription of interleukin-2 (IL-2), thus inducing T cell proliferation. The calcium-activated potassium channel KCa3.1 and the voltage-dependent Kv1.3 alternately open during this process and give rise to the potassium efflux that hyperpolarizes the plasma membrane, thus providing the driving force for sustained Ca^2+^ influx (Teisseyre et al., 2019). This figure was created using images from Servier Medical Art (http://smart.servier.com). Servier Medical Art by Servier is licensed under a Creative Commons Attribution 3.0 Unported License.

Inhibition of other Kv channels by specific toxins was also shown to reduce cancer cell proliferation and tumor size. In general, many studies addressed the relevance of Kv1.3 for proliferation in different cancer lines (e.g., Abdul et al., 2003; Preussat et al., 2003; Wu J. et al., 2013) or in primary cells (e.g., Smith et al., 2002; Grossinger et al., 2014; Petho et al., 2016). The proposed mechanism linking Kv1.3 function and its ability to set membrane potential to proliferation is summarized in Figure 2 for the case of lymphocytes. The same proliferation-promoting mechanism may apply for pathological lymphocytes, where upregulation of Kv1.3 ensures a continuous driving force for calcium entry into the cells. Margatoxin (MgTx), an inhibitor of Kv1.3 (but also of Kv1.1 and Kv1.2; Bartok et al., 2014), when injected directly into the xenograft nude mice model of A549 human lung adenocarcinoma at 1 nM concentration, significantly inhibited tumor growth. This was proposed to be ascribable to the effect of MgTx on cell cycle progression (Jang et al., 2011a). In addition to cancer, Kv1.3 activity has been correlated to the proliferation of many other types of cells, including vascular smooth muscle cells (Cidad et al., 2010; Cheong et al., 2011). Kv1.3 blockade with PAP-1 decreased the proliferation rate of these cells by downregulating receptor tyrosine kinases and the cell cycle regulator Early Growth Response-1 (EGR1) (Lasch et al., 2020). Further, a recent report highlighted that the cajanine derivative LJ101019C (1 μM), able to enhance Kv1.3 activity and expression, leads to natural killer (NK) cell proliferation and activation *via* AKT/mammalian Target Of Rapamycin (mTOR) signaling (Geng et al., 2020), representing thus a promising candidate for NK-based immunotherapy against cancer.

As to Kv1.5, this channel has been shown to be upregulated in many types of tumors and metastatic tissues and promotes proliferation. However, expression of Kv1.5 shows an inversed correlation with malignancy in some gliomas and non-Hodgkin’s lymphomas (Comes et al., 2013, 2015), while its high expression, along with that of Kv1.3, correlates with leiomyosarcoma proliferation and aggressiveness (Bielanska et al., 2012). In accordance, silencing Kv1.5 expression in osteosarcoma significantly inhibited proliferation and induced a cell cycle arrest at G0/G1 phase (Wu et al., 2015) and channel expression has been linked to proliferation in several works (see e.g., Vallejo-Gracia et al., 2013; Chow et al., 2018).

**FIGURE 3:**
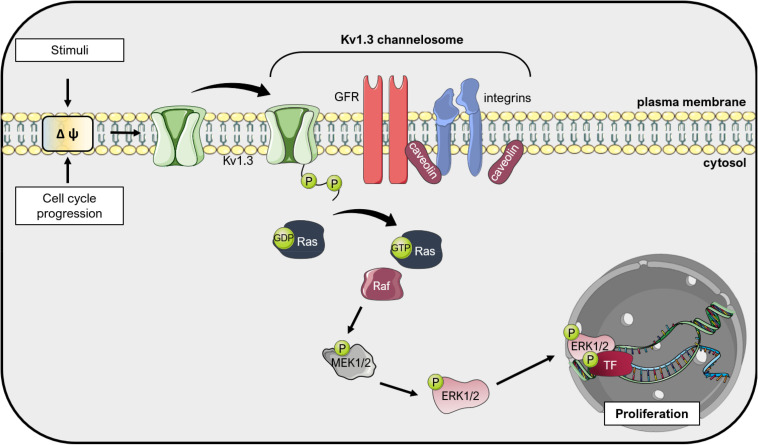
Kv1.3 regulates cell proliferation independently from ion conductance: the «voltage sensor model». Changes in the plasma membrane potential, that occur during cell cycle progression or upon application of external stimuli, induce a conformational change in Kv1.3 due to its voltage-sensor domain. Because Kv1.3 interacts with other proteins in large complexes (the so-called Kv1.3 channelosome), these conformational changes translate into phosphorylation of the C-terminus of Kv1.3 and activation of pro-proliferative signal transduction cascades, including integrin and growth factor receptor (GFR) signaling. These events result in activation of Ras, which activates the Raf/Mitogen-activated protein kinase kinase (MEK)/Extracellular signal-Regulated Kinase (ERK) pathway, finally leading to proliferation mediated by different downstream transcription factors (TF) such as c-Myc (Pérez-Garcìa et al., 2018). This figure was created using images from Servier Medical Art (http://smart.servier.com). Servier Medical Art by Servier is licensed under a Creative Commons Attribution 3.0 Unported License.

Altogether, a conclusive general picture regarding the involvement of Kv1.1, Kv1.3, and Kv1.5 in promoting cancer cell proliferation in different types of cancers where these channels are expressed is still lacking, such as the exact mechanism(s) involved in channel-mediated signaling leading to proliferation. These proteins can apparently promote proliferation also independently of their ion-conducting properties, but in function of the presence of two phosphorylation sites at the C-terminus of the channels (Figure 3; see e.g., Cidad et al., 2012; Jimenez-Perez et al., 2016). Only sporadic studies addressed the role of other Kv1. channels in proliferation (see e.g., Vautier et al., 2004).

### Kv2 Channels

Among channels of the Kv2. family, Kv2.1 (in heteromers with the silent subunit Kv9.3) seems to be the major component of Kv channels in cervical adenocarcinoma cells. Silencing of the Kv2.1 partner Kv9.3 using small interfering RNA caused G0/G1 cell cycle arrest in colon and lung carcinoma cell lines (Lee et al., 2015). The specific blocker of Kv2.1 Hanatoxin-1 significantly reduced proliferation of different cell lines by up to 40% (Suzuki and Takimoto, 2004). Another study showed that Ts15, a toxin from *Tityus serrulatus*, inhibited proliferation of central memory T cells, where Kv2.1 is highly expressed (Pucca et al., 2016), similarly to the hippocampus. In contrast, downregulation of Kv2.1 expression in HEK293 cells by Tacrine, a cholinesterase inhibitor, was able to boost proliferation (Hu X.M. et al., 2020). To our knowledge, Ts15 and Hanatoxin-1 have not been explored in the context of cancer in other studies.

### Kv3 Channels

It has been reported that block of Kv3.1-specific currents or genetic ablation of the channel inhibits proliferation and migration of oligodendrocyte progenitor cells (Tiwari-Woodruff et al., 2006), while block of Kv3.4 resulted in a reduced proliferation rate of vascular smooth muscle cells (Miguel-Velado et al., 2005, 2010). Kv3.1 and Kv3.4 both contribute to lung adenocarcinoma and breast cancer cell migration and tumor invasiveness, although their inhibition did not affect cell proliferation (Song et al., 2018). On the other hand, a recent report elucidated the mechanism by which Kv3.4 channel is involved in oral squamous cell carcinoma growth (Qian et al., 2019). In this study, hypoxia was found to induce the Hypoxia-Inducible Factor (HIF-1α), which mediates increased Kv3.4 expression, proliferation, migration and invasion of oral squamous cell carcinoma cells. Kv3.4 also plays a role in mediating radio-resistance of myeloid leukemia cells by increasing its activity after application of ionizing radiation and inducing a G2/M arrest due to hyperpolarization of the membrane, calcium influx, Calcium/calmodulin-dependent protein Kinase (CamKII) activation and following inactivation of the phosphatase cdc25B and the cyclin-dependent kinase cdc2, both important for the G2/M transition (Palme et al., 2013).

### Kv4 Channels

Kv4 channels have been characterized mainly in the context of neuronal function, but a recent work identified peptidyl-prolyl cis-trans isomerase NIMA-interacting 1 (Pin1), a prolyl isomerase that promotes cancer cell proliferation, as an interactor of Kv4.2 (Hu J.H. et al., 2020). The importance of this interaction was underlined in the context of cognitive flexibility, but not of cancer so far.

### Kv7 Channels

The Kv7 channels are encoded by *KCNQ* genes and are expressed in cardiac myocytes, smooth muscle cells, neurons, and epithelial cells, where they play a role in various physiological processes (González et al., 2012), including that of regulating proliferation (Roura-Ferrer et al., 2008). A recent work highlighted Kv7.5 as promising therapeutic target for vascular tumors, as its expression clearly correlated with neoplastic malignancy (Serrano-Novillo et al., 2020). Interestingly, Tamoxifen, often used against breast cancer, inhibits Kv7.2 and Kv7.3 that are the main ion channels contributing to the so-called M-current, which regulates neuronal excitability (Ferrer et al., 2013). However, the role of this effect in the context of breast or other cancers has not been explored.

### Kv10 and Kv11 Channels

In addition to the above-mentioned channels, work from different labs on Kv10.1 and Kv11.1 indicated their important role in cancer development and progression. The Kv10.1 channel was the first with proven oncogenic potential (Pardo et al., 1999) and is overexpressed in about 70% of human tumor biopsies (Urrego et al., 2014). Kv10.1 was proposed to be crucially involved in the resorption of the primary cilium during the G2/M phase of the cell cycle, thus favoring cell cycle progression (Urrego et al., 2017). As to Kv11.1, this channel has been associated to different tumoral processes such as cell cycle progression, angiogenesis, invasiveness and metastasis formation and the channel is also overexpressed in a variety of cancer cells with respect to corresponding non-cancer tissues (He S. et al., 2020). Indeed, implications of Kv11.1 on cell cycle and proliferation in gastric, pancreatic and breast cancer have already been established, and the channel was shown to influence tumor progression of esophageal squamous cell carcinoma, bladder cancer and osteosarcoma as well (Zeng et al., 2016; Xu et al., 2018; Wang et al., 2019). Recent results revealed that in esophaegal squamous cell carcinoma, Kv11.1 stimulates Phosphatidylinositol 3-Kinase (PI3K)/Akt, which leads to upregulation of thioredoxin domain-containing protein 5 (TXNDC5), thus increasing proliferation, inhibiting apoptosis and favoring epithelial-to-mesenchymal transition. Kv11.1 knockdown accordingly reduced tumor growth and metastasis formation *in vivo* (Wang et al., 2019). In bladder cancer, miR-96 was found to regulate Kv11.1 expression. Using a miR-96 inhibitor, Kv11.1 expression decreased, and cells were arrested at the G1 phase. miR-96 inhibition further increased apoptosis and decreased migration of bladder cancer cells (Xu et al., 2018). Similar effects on proliferation, migration and apoptosis were obtained in osteosarcoma cells by inhibition of Kv11.1, which reduced Nuclear Factor kappa-light-chain-enhancer of activated B cells (NF-κB) activity through decreased PI3/Akt signaling (Zeng et al., 2016).

## Voltage-Gated Potassium Channels and Regulation of Programmed Cell Death

Strikingly, most of the above-mentioned channels are intimately linked not only to proliferation, but also to the regulation of apoptosis (Szabo et al., 2010). Here, we discuss the role of Kv channels in cell death.

### Kv1 Channels

Kv1.3 was one of the first ion channels whose function was shown to be modulated under various phases of apoptosis. In particular, the channel was shown to be inhibited within a few minutes following apoptosis induction by CD95 (Szabo et al., 1996) or by ceramide (Gulbins et al., 1997), due to tyrosine phosphorylation of the channel. This post-translational modification was shown to inhibit Kv1.3 activity also in other contexts (Holmes et al., 1996; Cayabyab et al., 2000). On the other hand, in a still early phase of apoptosis, the channel was shown to be activated by apoptotic stimuli and to contribute to the so-called apoptotic volume decrease in lymphocytes (Storey et al., 2003) that is associated with the activation of the apoptotic machinery (Bortner and Cidlowski, 2004, 2014). Further work is required to understand the importance of these findings, since Margatoxin, an inhibitor of Kv1.3, neither induced nor inhibited apoptosis in these studies. Nonetheless, using genetic models or silencing of Kv1.3, it was clearly shown that Kv1.3 expression is required for apoptosis (Bock et al., 2002), even in primary cells (Szabo et al., 2008), since in the absence of the channel the cells turned apoptosis-resistant. This finding might explain why the channel is found less expressed in several types of cancer tissue samples at advanced stage with respect to healthy tissues (Serrano-Novillo et al., 2019). Similar findings were reported for Kv1.1 in the same study. As a follow up of these findings, Kv1.3 was identified as functional channel in mitochondria (Szabo et al., 2005) and later studies identified the mitochondrial counterpart of the channel as a crucial player for intrinsic apoptosis (Szabo et al., 2011; Leanza et al., 2012a, 2017).

In addition to Kv1.3, other Kv1 channels have been linked to the apoptotic cascade (Szabo et al., 2010; Shah and Aizenman, 2014). For example, enhanced Kv1.1 expression protected hippocampal neurons against staurosporine- or glutamate-induced apoptosis (Lee et al., 2003; Shen et al., 2009). In contrast, downregulation of specifically Kv1.1 and of Kv1.3 expression in retinal ganglion cells (but not of Kv1.2 and Kv1.5) rescued neurodegeneration of these cells. In agreement, Margatoxin reduced cell death in this system, where Kv1.1 depletion was shown to increase the expression of the antiapoptotic Bcl-X_L_, while depletion of Kv1.3 reduced the pro-apoptotic caspase-3, caspase-9 and Bad (Koeberle et al., 2010). Thus, also in the case of Kv1.1, further work is required to understand the different contribution of this channel to degenerative death in distinct cell types.

Since Kv1.5 is overexpressed in many types of cancer cells (Comes et al., 2013), it represents a good target in this context. Indeed, the role of Kv1.5 in apoptosis has been extensively studied. Bonnet and colleagues correlated low Kv1.5 expression in cancer cells to apoptosis resistance (Bonnet et al., 2007). In particular, these authors observed that metabolic shift toward oxidative phosphorylation and the consequent reactive oxygen species release from mitochondria activates plasma membrane Kv1.5 with a resulting reduction of proliferation and induction of apoptosis. On the other hand, a more recent work showed that silencing Kv1.5 expression in osteosarcoma cells not only reduces proliferation by blocking the cell cycle at G0/G1 phase, as expected, but also enhances apoptosis through upregulation of Bax and caspase-3 and downregulation of anti-apoptotic Bcl-2 and Bik (Wu et al., 2015). Pharmacological inhibition of Kv1.5 by diphenyl phosphine oxide-1 (DPO-1) contributed to apoptosis induction in macrophages (Leanza et al., 2012b). In another study however, silencing of Kv1.5 with siRNA reduced palmitate-induced endothelial apoptosis (Du et al., 2017). In a recent investigation, inhibition of Kv1.5 by DPO-1 prevented the mitochondria-dependent apoptosis-triggering effect of Apigenin, a dietary flavonoid, in pulmonary artery smooth muscle cells (He Y. et al., 2020). Similarly, DPO-1 reduced hydrogen peroxide-evoked endothelial cell apoptosis (Chen et al., 2012).

In summary, while considerable experimental work links plasma membrane Kv channels to apoptosis, contrasting findings about their expression level in cancer cells as well as their involvement in the regulation of the apoptotic pathway deserves attention. While expression levels might change depending on the cancer stage (with downregulation of the channels associated with apoptosis-resistance in advanced stage and/or metastatic cells), the different outcome on apoptosis might be tentatively explained by modulation of channel activity by yet-undefined factors such as protein interaction partners, whose nature might change depending on the type/stage of cancer.

### Kv2 Channels

**FIGURE 4:**
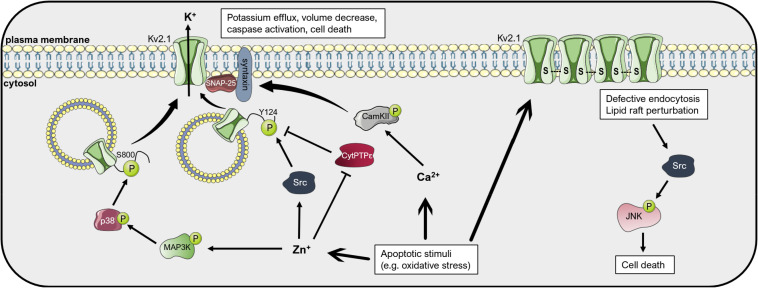
Mechanisms of Kv2.1-mediated cell death. Apoptotic stimuli such as oxidative stress lead to an increase in cytosolic free Zn^2+^ and Ca^2+^ concentrations. Zn^2+^ induces the activation of the kinases p38 and Src, which phosphorylate the intracellular pool of Kv2.1 at the residues Ser800 and Tyr124, respectively. These phosphorylations lead to insertion of Kv2.1 in the plasma membrane, an increase in the outgoing K^+^ current, apoptotic volume decrease and cell death. Plasma membrane insertion of the channel depends on interaction with the t-SNARE proteins SNAP-25 and syntaxin. The latter interacts with CamKII, activated upon an increase in Ca^2+^, favoring Kv2.1 localization to the membrane. Kv2.1 can induce apoptosis also in an ion-conducting-independent manner. Oxidative stress favors oligomerization of the protein by formation of disulfide bridges, which leads to defective endocytosis and lipid raft perturbation, resulting in activation of the Src-JNK signaling axis, finally inducing apoptosis. Please refer to the text for references and additional details. This figure was created using images from Servier Medical Art (http://smart.servier.com). Servier Medical Art by Servier is licensed under a Creative Commons Attribution 3.0 Unported License.

The Kv2.1 channel has been extensively studied in the context of neuronal apoptosis (Figure 4). An involvement of Kv2.1 in the initiation of programmed cell death has already been proposed in 2001, when Ekhterae and colleagues found that an overexpression of Bcl-2 in rat pulmonary artery smooth muscle cells inhibited staurosporin-induced apoptosis by downregulating Kv1.1, Kv1.5, and Kv2.1 expression and reducing the potassium efflux that initiates the apoptotic volume decrease (Ekhterae et al., 2001). The importance of Kv2.1 for programmed cell death of cortical neurons was first shown by Pal and co-workers. They demonstrated that the expression of dominant-negative Kv2.1 in cortical neurons abolished the potassium efflux that characterizes the onset of apoptosis and increased the resistance to apoptotic stimuli induced by the oxidant 2,2′-dithiodipyridine (DTDP) or staurosporine. Accordingly, the transient expression of functional Kv2.1 in Chinese hamster ovary (CHO) cells increased the susceptibility to DTDP treatment and caspase-dependent cell death (Pal et al., 2003). The same group identified compound 48F10 that inhibits Kv2.1 in the low micromolar range and confers resistance to DTDP treatment in cortical neurons and enterocytes (Zaks-Makhina et al., 2004; Grishin et al., 2005). Upon application of apoptotic stimuli, an increased trafficking of Kv2.1 to the plasma membrane is responsible for the augmented outgoing current. The *de novo* insertion of Kv2.1 channels in the cell membrane depends on syntaxin and synaptosomal-associated protein (SNAP-25), proteins belonging to the SNAP receptor (t-SNARE) complex, normally responsible for exocytotic neurotransmitter release (Pal et al., 2006; Yao et al., 2009; McCord et al., 2014). The interaction of Kv2.1 with syntaxin and its insertion in the plasma membrane upon oxidative stress signals also requires the action of CamKII. Calcium thus emerges as a regulator of the potassium current surge at the onset of neuronal apoptosis (McCord and Aizenman, 2013). Interestingly, targeting the Kv2.1-syntaxin interaction with a peptide mimicking the syntaxin-1A binding domain of Kv2.1 ameliorated cell death in an *in vivo* model of ischemic stroke (Yeh et al., 2017).

Membrane trafficking of Kv2.1 is regulated by multiple phosphorylation events (Figure 4). For example, activation of the mitogen-activated protein (MAP) kinase p38 is necessary for the induction of neuronal apoptosis after oxidative injury. By phosphorylating a serine residue at the C-terminal of the channel protein (S800), active p38 leads to membrane insertion of Kv2.1 and the potassium current surge that initiates cell death, possibly by changing the association of Kv2.1 with other proteins (such as syntaxin) (Redman et al., 2007). p38 activation, in turn, depends on an intracellular zinc increase caused by oxidants such as peroxynitrite (Zhang et al., 2004). Intracellular zinc leads, in addition to p38 activation, also to inhibition of the phosphatase Cyt-PTPε that in standard conditions dephosphorylates Kv2.1 at a tyrosine residue at the N-terminal (Y124). Src kinase is responsible for phosphorylation of the channel at Y124, enhancing the Kv2.1-mediated current at the onset of apoptosis (Redman et al., 2009). Phosphorylation at S800 and Y124 mutually co-regulate each other, with P-Y124 facilitating the action of p38 on S800 and *vice versa* (He et al., 2015). Additionally, serum deprivation, which induces apoptosis of cortical neurons in a model of excitotoxicity, leads to N-Methyl-d-aspartate (NMDA) receptor activation and enhanced plasma membrane expression of Kv2.1 due to dephosphorylation of the channel protein by the Protein Phosphatase 1 (PP1) and/or 2A (PP2A) (Yao et al., 2009). It was further shown that activation of Protein Kinase A (PKA) following an increase in cyclic AMP protects cerebellar granular neurons from cell death induced by potassium-low and serum-free medium (Jiao et al., 2007). In this study, PKA activation decreased the Kv2.1-dependent outgoing potassium current upon application of the pro-apoptotic stimulus, likely by reducing Kv2.1 expression. Cleavage of Kv2.1 can also affect cell death induction. Liu et al. recently showed that beta-secretase 2 (BACE2), a protease that frequently shows increased expression in Alzheimer’s disease, cleaves Kv2.1 at three different sites, reducing the current surge at the onset of apoptosis and protecting neurons from cell death (Liu et al., 2018).

The complex interplay between Kv2.1 and cell death plays an important role in different diseases. In many central nervous system (CNS) disorders, microglia are involved in promoting neurodegeneration. The mechanisms described above partly account for the neurodegenerative effect of microglia: Knoch et al. (2008) showed that activated microglia release reactive oxygen and nitrogen species, which increase the intracellular zinc concentration in co-cultured neurons, finally leading to p38 activation, a surge in Kv2.1-mediated potassium currents and induction of cell death. In addition to neurodegeneration, p38 activation and apoptosis induction play a relevant role also in hepatitis virus C (HVC) infections. The viral protein NS5A blocks oxidative stress-mediated p38 activation by directly binding and blocking the upstream MAPK kinase kinase MLK3, thus effectively inhibiting apoptosis initiation via Kv2.1 and favoring the survival of HCV-infected hepatoma cells even in the presence of pro-apoptotic stimuli (Mankouri et al., 2009; Amako et al., 2013). Interestingly, also expression of NS5A1b in neurons is protective as it inhibits Kv2.1-mediated apoptosis, although in a Src-dependent manner (Norris et al., 2012). Human immunodeficiency virus 1 (HIV-1) is involved in neuronal apoptosis as well. Its envelope glycoprotein gp120 has been associated to the pathogenesis of HIV-1-associated neurodegenerative disorders by acting on Kv2.1. In hippocampal neurons, gp120 was shown to increase Kv2.1 expression and current density in a p38- and caspase-3-dependent manner, thus enhancing apoptosis (Shepherd et al., 2012, 2013; Liu et al., 2013; Zhu et al., 2015). p38 plays a role also in methamphetamine-induced neuronal damage. This addictive drug exhibits strong neurotoxicity, inducing apoptosis *via* activation of p38, upregulation of Kv2.1 and cleavage of caspase-3 (Zhu et al., 2018).

Oxidative stress has been associated to different conditions that involve neuronal damage, including Alzheimer’s (AD) and Parkinson’s disease (PD), aging, amyotrophic lateral sclerosis and stroke (Radi et al., 2014). It has been shown that carbon monoxide (CO) protects neurons from oxidant-induced apoptosis by inhibition of Kv2.1, and that this inhibition is in part exerted by mitochondrial reactive oxygen species (ROS) (Dallas et al., 2011). Kv2.1 inhibition exerted by CO may also play a relevant role in the resistance mechanism against oxidative stress of medulloblastoma cells. In fact, the hypoxic environment of tumors favors the constitutive expression of heme oxygenase 1 (HO-1), which catalyzes heme and releases CO as a byproduct, finally leading to Kv2.1 inhibition and apoptosis resistance (Al-Owais et al., 2012). Interestingly, Donepezil, an acetylcholinesterase inhibitor used in AD, was shown to inhibit Kv2.1 with an IC_50_ value of 7.6 μM and may thus add protection against neuronal cell death (Yuan et al., 2011). Direct oxidation of Kv2.1 has also been proposed as a mechanism of neurotoxicity (Figure 4). Cotella et al. (2012) found in both *in vitro* and *in vivo* settings that an oxidative environment leads to channel oligomerization at the plasma membrane due to the formation of disulfide bridges between channel subunits. These oligomers, although exhibiting decreased open probability, triggered apoptotic cell death. Kv2.1 oligomerization was greatly increased in the brain of a mouse model of AD. In addition, application of amyloid-β to cultured cells induced the formation of oligomers and cell death (Cotella et al., 2012). Kv2.1 oligomerization resulted in defective endocytosis, lipid raft perturbation and stress-activated c-Src/c-Jun N-terminal kinase (JNK) signaling which finally initiated apoptosis. Cholesterol, by stabilizing lipid rafts, could revert the onset of apoptosis (Wu X. et al., 2013). Accordingly, transgenic mice harboring a non-oxidizable Kv2.1 mutant showed decreased inflammation, neurodegeneration and cell death after traumatic brain injury and exhibited improved outcomes in cognitive and motor behavioral assays with respect to control mice (Yu et al., 2016). Importantly, the results from these experiments were mimicked by treatment of control mice with Dasatinib, a Src kinase inhibitor. The findings of these studies suggest that under oxidizing conditions, such as during aging or neurodegenerative diseases, Kv2.1 channels play a critical role in the regulation of cell death in a manner that may be independent from their ion-conducting properties.

In addition to its well-established role in neuronal apoptosis, Kv2.1 is involved in the cell death regulation of the neuroendocrine pancreatic β-cells. In these cells, Kv2.1 channel controls glucose-stimulated insulin secretion and death induction. The incretin hormones Gastric Inhibitory Peptide (GIP) and Glucagon-like peptide-1 (GLP-1) have been found to profoundly alter Kv2.1 posttranslational modifications, promoting the channel’s internalization and promoting cell survival in stress conditions (Kim et al., 2012). Additionally, the small-molecule inhibitors of Kv2.1 vindoline, SP6616 and ETA were shown to improve β-cell dysfunction and survival and ameliorate hyperglycemia *in vivo*, suggesting that Kv2.1 inhibition may be exploited in anti-diabetic therapies (Yao et al., 2013; Zhou et al., 2016, 2018).

### Kv3 and Kv4 Channels

Similarly to Kv2.1, some reports assign a role in the regulation of neuronal apoptosis to Kv3.4. For example, amyloid-β has been found to increase both Kv3.4 expression and activity in hippocampal neurons through NF-κB and to induce cell death, an effect that could be reverted by a pan-Kv3 inhibitor (Pannaccione et al., 2007). Similarly, Kv3.4 downregulation exerted by HIF-1α under oxidative stress reduced the potassium current and exhibited a neuroprotective effect in the neuroblastoma cell line SH-SY5Y (Song et al., 2017). Interestingly, the authors of this study suggest an involvement of mitochondrial Kv3.4 in the regulation of oxidative stress-induced neuronal damage. Amyloid-β peptides were also found to increase the expression of Kv4.2 and Kv4.3 channels, leading to increased potassium currents and apoptosis of cerebellar granule cells (Pieri et al., 2010).

### Kv7 Channels

Regarding Kv7 channels, their inhibition was demonstrated to trigger spiral ganglion neuron death, possibly underlying the autosomal dominant version of progressive hearing loss associated with Kv7.4 mutations (Lv et al., 2010). On the other hand, activation of Kv7.2 and Kv7.3 in hippocampal neurons induced Extracellular signal-Regulated Kinase (ERK) 1/2 activation and caspase-3 cleavage, an effect that could be prevented by the pan-Kv7 blocker XE991 (Zhou et al., 2010). Interestingly, blockade of Kv7 channels by amyloid precursor proteins may regulate neuronal excitation and possibly cell death (Lee et al., 2020).

### Kv10 and Kv11 Channels

Voltage-gated potassium channels belonging to the ether-à-go-go (EAG) family are also involved in the regulation of cell death. This family comprises three subgroups, namely EAG (aka Kv10), Eag-Related Gene (ERG, aka Kv11), and Eag-Like (ELK, aka Kv12). While Kv10 and Kv12 channels are expressed primarily in the central nervous system, Kv11 is present also in the heart, where it regulates the termination of the cardiac action potential, and in smooth muscle tissues (Barros et al., 2020). Ion channels belonging to the EAG family have been intensively studied in the context of cancer. Their expression is frequently high in a wide range of tumor types, while lacking in the corresponding non-tumoral tissues (He S. et al., 2020). Although, to our knowledge, in-depth mechanistic studies regarding the involvement of EAG channels in the regulation of apoptosis are missing, different studies suggest that channel activity may either promote or block the induction of cell death depending on the cell type and the environment. Studies in which targeting Kv10 or Kv11 channels was exploited to induce tumor cell death will be analyzed more in detail below.

## Targeting Voltage-Gated Potassium Channels in Cancer

Although several types of Kv channels are linked to either altered proliferation, or to apoptosis induction, concrete steps toward cancer treatment have been obtained only in few cases. This situation is most probably due to our limitation of having promiscuous pharmacological inhibitors as tools to modulate the function of these channels. Nonetheless, the use of toxins, small molecules and specific antibodies is emerging as promising strategy (for recent review see e.g., Wulff et al., 2019; Díaz-García and Varela, 2020).

### Kv1 Channels

Intratumoral injection of Margatoxin, a Kv1.3 inhibitor acting on the plasma membrane Kv1.3, reduced lung cancer volume *in vivo* (Jang et al., 2011a), while intraperitoneal injection of PAP-1, the small molecule inhibitor of this channel, did not reduce tumor volume in an orthotopic melanoma model (Leanza et al., 2017; Peruzzo et al., 2020). Two toxins, acting on Kv1.1 (KAaH2) and Kv1.3 (KAaH1 that acts on Kv1.1 as well) derived from the *Androctonus australis* Hector venom, were shown to inhibit glioma proliferation and migration, at least *in vitro* (Aissaoui et al., 2018). A disadvantage of these toxins, however, is their inability to cross the blood brain barrier and to act on intracellular potassium channels. Instead, recent evidence indicates that mitochondrial ion channels can be of importance in fighting cancer. For example, pharmacological inhibition of the mitochondria-located counterpart of Kv1.3 channel by specific mitochondria-targeted drugs was shown to trigger apoptotic death selectively in cancer cells *in vivo*, without inducing alterations to healthy cells and tissues (Leanza et al., 2017).

### Kv10.1

**FIGURE 5:**
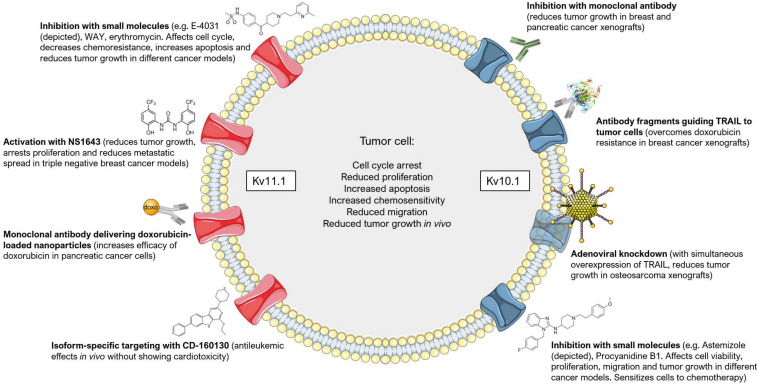
Targeting Kv10.1 and Kv11.1 in cancer. Different approaches have been employed to target Kv10.1 (on the right) and Kv11.1 (on the left) in tumor models. The most common effects that are induced by channel inhibition, activation (in the case of Kv11.1) or downregulation are summarized in the center. Please refer to the text for additional details and references. This figure was created using images from Servier Medical Art (http://smart.servier.com). Servier Medical Art by Servier is licensed under a Creative Commons Attribution 3.0 Unported License.

Different strategies have been exploited to specifically target Kv10.1 and Kv11.1 channels in order to either block proliferation of cancer cells or to induce their apoptosis, as summarized in Figure 5. Given the structural similarity between the potassium channel superfamily, specific blockers are difficult to obtain, therefore some of the research has focused on antibody-based or genetic strategies. For example, a monoclonal antibody raised against Kv10.1 dose-dependently inhibited Kv10.1 currents in neuroblastoma cells, decreased the proliferation of different human cancer cell lines and reduced tumor growth in xenograft models of breast and pancreatic cancer, notably, without acting on Kv10.2 or Kv11.1 (Gomez-Varela et al., 2007). Additionally, specific small antibody fragments targeting the extracellular pore domain of Kv10.1 were exploited to selectively guide the Tumor necrosis factor-Related Apoptosis-Inducing Ligand (TRAIL) to tumor cells. These constructs sensitized breast cancer cells to chemotherapeutics they were otherwise resistant to Hartung and Pardo (2016), or directly induced cell death in prostate and pancreatic cancer cells when fused to a TRAIL variant with enhanced pro-apoptotic activity (Hartung et al., 2020). This antibody-based strategy was proven efficacious also *in vivo* (Hartung and Pardo, 2016). The specificity of this treatment relies on the enhanced expression of Kv10.1 in many cancer types (Pardo et al., 1999; Pardo and Stuhmer, 2014) and the tumor selectivity of TRAIL. Similarly, treating the human osteosarcoma cell line MG-63 or mice bearing an osteosarcoma xenograft with adenoviral vectors that simultaneously knockdown Kv10.1 and overexpress TRAIL led to tumor regression and apoptosis of cancer cells (He et al., 2013).

Another strategy for the treatment of cancer that has gained increased interest in recent years is drug repositioning using clinically safe drugs already exploited for some other disease. Regarding Kv10.1, the anti-histaminic drug Astemizole was found to inhibit this channel with an IC_50_ value of only 200 nM (García-Ferreiro et al., 2004). This compound was associated with a reduced cancer mortality by sensitizing cells to chemotherapy (Ellegaard et al., 2016). It reduced, for instance, proliferation and viability of keratinocytes expressing the human papilloma virus (HPV) oncogenes E6/E7, that mimic cervical cancer cells and express Kv10.1 (Diaz et al., 2009). The same drug was used to target proliferation and viability of hepatocellular carcinoma cells *in vitro* and in an *in vivo* mouse model, where it decreased Kv10.1 expression (de Guadalupe Chávez-López et al., 2015). Furthermore, Astemizole increased the anti-proliferative effects of calcitriol on breast cancer both *in vitro* and *in vivo* by acting also on Kv10.1 (García-Quiroz et al., 2012, 2014) and increased the sensitivity of lung cancer cell lines to the EGFR inhibitor Gefitinib possibly by acting on both Kv10.1 subcellular localization and expression (de Guadalupe Chávez-López et al., 2017). Knockdown of Kv10.1 or its inhibition by Astemizole sensitized glioblastoma cells to Temozolomide treatment, and SH-SY5Y cells to rotenone-induced apoptosis (Sales et al., 2016; Horst et al., 2017). Astemizole thus represents a valid candidate for drug repositioning (Figure 5).

Natural compounds are also promising tools. Very recently, the natural compound Procyanidin B1 was identified as a rather specific inhibitor of Kv10.1. It reduced the potassium current with an IC_50_ value of 10 μM, suppressed proliferation and migration of hepatoma cells and inhibited tumor growth *in vivo* in a xenograft model of liver cancer (Na et al., 2020). In addition, the only Kv10.1-specific toxin, k-Hefutoxin 1 from *Heterometrus fulvipes* scorpion venom (Moreels et al., 2017), might be useful in the context of cancer therapy, but to our knowledge no attempts have been made so far to test its *in vitro* and *in vivo* effects (Díaz-García and Varela, 2020).

### Kv11.1

Although representing an interesting target for cancer therapy, Kv11.1 inhibitors have limited use in the clinic because they are potentially cardio-toxic. Kv11.1 is expressed in the heart, where it regulates the cardiac action potential, and the use of channel blockers has been associated with cardiac arrhythmia (extensively reviewed in Arcangeli and Becchetti, 2010). Nonetheless, some studies achieved promising results with channel activators or blockers (Figure 5). For example, the incidental administration of FDA-approved drugs with proven inhibitory activity on Kv11.1 increased the survival of glioblastoma patients that showed a high expression of Kv11.1 (Pointer et al., 2017). These drugs did not have any adverse side-effect on cardiac activity and include Phenytoin, Haloperidol, Fluoxetine, Tamoxifen, Amitriptyline, and Ketoconazole (Pointer et al., 2017). In acute lymphoblastic leukemia (ALL) cells, the Kv11.1 inhibitors E-4031, WAY123,398 and erythromycin (which, importantly, shows no cardiotoxicity) decreased the chemoresistance of these cells and increased the pro-apoptotic effect of the chemotherapeutics Doxorubicin, Prednisone and Methotrexate. E-4031 significantly reduced tumor progression and improved survival also *in vivo* (Pillozzi et al., 2011). Simultaneous activation of the calcium- and voltage-dependent potassium channel KCa3.1 with SKA-31 and inhibition of Kv11.1 with E-4031 further sensitized colorectal cancer cells to cisplatin treatment both *in vitro* and *in vivo* (Pillozzi et al., 2018). Erythomycin was also shown to increase the cytotoxic effect of paclitaxel, vincristine and hydroxy-camptothecin on different cancer cell lines that express Kv11.1 and induce an arrest in the G2/M phase (Chen et al., 2005). Similarly, the gastroprokinetic drug cisapride was shown to inhibit Kv11.1 at low nanomolar concentrations and, accordingly, arrested cell growth and increased apoptosis in Kv11.1-expressing gastric cancer cell lines (Shao et al., 2005). Further, inhibition of Kv11.1 by doxazosin led to apoptosis and cell cycle arrest in the G0/G1 phase of glioblastoma cells. The effect of doxazosin was mimicked by siRNA-mediated knockdown of the channel (Bishopric et al., 2014).

Interestingly, two main isoforms are known for Kv11.1, namely Kv11.1A and Kv11.1B. While Kv11.1A is expressed predominantly in the heart, Kv11.1B is the prevalent isoform in leukemia. This peculiarity has been exploited by Gasparoli et al. (2015), that characterized compound CD-160130, a small-molecule inhibitor of Kv11.1 that does not cause cardiac arrhythmia, possibly by blocking isoform B with a higher efficiency than isoform A (Figure 5). CD-160130 caused apoptosis and growth arrest of leukemic cells *in vitro* and showed strong antileukemic effects *in vivo* (Gasparoli et al., 2015). The higher expression of Kv11.1 in cancer cells with respect to healthy tissues (Pardo and Stuhmer, 2014) was also exploited by Spadavecchia and colleagues. They engineered polyethylene-glycol gold nanoparticles fused to an anti-Kv11.1 antibody to selectively deliver doxorubicin to pancreatic cancer cells (Spadavecchia et al., 2016). In addition, the antibiotic clarithromycin, whose mammalian targets include Kv11.1, was shown to induce apoptotic cell death and increase the cytotoxic effects of 5-fluorouracil both *in vitro* and *in vivo* in colorectal cancer models (Petroni et al., 2020). Finally, to avoid cardiac side-effects, Gentile and co-workers proposed an opposite approach to Kv11.1 channel blockers. They used a small-molecule activator of the channel, NS1643, to achieve *in vivo* inhibition of tumor growth of triple-negative breast cancer (Fukushiro-Lopes et al., 2017; Figure 5). In their model, prolonged activation of the channel leads to hyperpolarization, increase in ROS production, DNA damage, activation of a senescence program and arrest of proliferation. Importantly, NS1643 did not cause cardiac arrythmias. In a subsequent study, they showed NS1643 to reduce the metastatic spread *in vivo* in breast cancer models and to reprogram epithelial-to-mesenchymal transition and cancer stemness of MDA-MD-231 cells by reducing Wnt/β-catenin signaling (Breuer et al., 2019). However, possible side effects of NS1643, other than cardiotoxicity, have not been investigated in a systematic way *in vivo.*

In addition, toxins might offer a way of intervention: CsEKerg1 toxin, from the *Centruroides sculpturatus* scorpion, inhibits Kv11.1 currents in neuroblastoma cells, although with relatively low affinity (Nastainczyk et al., 2002). Unfortunately, this toxin has not been tested on brain-derived tumor cell proliferation (Díaz-García and Varela, 2020).

## Conclusion and Future Outlook

Collectively, these studies demonstrate that several ion channels are attractive targets for cancer therapy and regulation of the immune response. As outlined in the present review, many channels share at least some functional aspects with other channels and it might be necessary to define combinations of drugs acting on ion channels for successful tumor treatment. In addition, many channels are expressed in several cell types and it remains to be defined whether channels can be selectively targeted in tumor cells. Altogether, taking into account the information reported here, Kv10 and Kv11 family members seem to represent the most promising target among the plasma-membrane located voltage-gated potassium channels, although the issues of specificity and possible cardiotoxicity warrants caution for Kv10- and Kv11-targeting drugs, respectively. Finally, most channels that were studied so far localize to the plasma membrane or mitochondria, but it is certainly possible that ion channels in other organelles, for instance lysosomes, also play an important role in tumor cell growth or can be exploited as novel targets for tumor therapy in the future.

## Author Contributions

MB, IS, and EG: writing of the manuscript. WL, ME, SAA, and SP: commenting and editing. All authors contributed to the article and approved the submitted version.

## Conflict of Interest

The authors declare that the research was conducted in the absence of any commercial or financial relationships that could be construed as a potential conflict of interest.
